# Cardiac Rehabilitation Early after Sternotomy Using New Assistive VR-Enhanced Robotic Exoskeleton—Study Protocol for a Randomised Controlled Trial

**DOI:** 10.3390/ijerph182211922

**Published:** 2021-11-13

**Authors:** Mihaela Mocan, Sonia Irina Vlaicu, Anca Daniela Farcaș, Horea Feier, Simona Dragan, Bogdan Mocan

**Affiliations:** 1 Department of Internal Medicine, University of Medicine and Pharmacy Iuliu Hațieganu Cluj-Napoca, 400012 Cluj-Napoca, Romania; vlaicusonia@gmail.com; 2 Department of Cardiology, University of Medicine and Pharmacy Iuliu Hațieganu Cluj-Napoca, 400012 Cluj-Napoca, Romania; ancafarcas@yahoo.com; 3 Department of Thoracic Surgery, Institute of Cardiovascular Diseases Timisoara, University of Medicine and Pharmacy Victor Babes, 300041 Timisoara, Romania; horea.feier@gmail.com; 4 Department of Cardiology, Clinic of Cardiovascular Prevention and Rehabilitation, University of Medicine and Pharmacy Victor Babes, 300041 Timisoara, Romania; simona.dragan@umft.ro; 5 Department of Industrial Engineering and Robotics, Technical University of Cluj-Napoca, 400020 Cluj-Napoca, Romania; bogdan.mocan@muri.utcluj.ro

**Keywords:** post-sternotomy cardiac rehabilitation, robotic exoskeleton, non-immersive virtual reality exergame

## Abstract

(1) Background and objective: Cardiac rehabilitation (CR) means delivering health education by structured exercises with the means of risk reduction, in a cost-effective manner. Well-conducted CR improves functional capacity, decreases re-hospitalization, and reduces mortality up to 25%. We bring to attention the protocol of a randomised control trial with the aim of validating the prototype of an assistive upper-body robotic exoskeleton system enhanced with a non-immersive virtual reality exergame (CardioVR-ReTone) in patients who undergone cardiac surgery. (2) Methods: Description of the CardioVR-ReTone system and the technical specification, followed by the group selection, randomization and evaluated variables. (3) Expected results: The primary outcome measurement is the modification of life quality at the end of the CR exercise training program. Secondary outcomes will encompass measurements of sternal stability, muscular activity, cardiac response to exercise, pain level and compliance/adherence to CR. (4) Conclusions: Implementing these novel features of the CardioVR-ReTone system, addressability, and efficacy of CR, so problematic in certain situations and especially in cardiac surgery, will be greatly facilitated, being independent of the skills and availability of the rehabilitation therapist.

## 1. Introduction

The burden of cardiovascular diseases (CVDs) is high, in 2015 being estimated 422.7 million cases that led to 17.92 million deaths [1]. Thus, CVDs are the main cause of mortality in the middle-aged population worldwide [2].

Cardiac rehabilitation (CR) means delivering health education by structured exercises by means of risk reduction, in a cost-effective manner. Well-conducted CR improves functional capacity, decreases re-hospitalization, as well as reducing mortality up to 25% [3]. A suitable rehabilitation is essential for enabling those people to live independently and enhance their quality of life (QoL) as they age. Most of these rehabilitation treatments need sessions of rehabilitation therapy to fortify muscles, to improve the motion of joints, to restore cardiac functional capabilities, and to globally increase freedom of movement [4,5]. In order to be time efficient, a CR therapy after open cardiac surgery should start in hospital and continue partly under supervision, partly at home, for 2 to 6 weeks [5,6]. The continuity of care is often interrupted in the transition from hospital to home due to the limited number of qualified human therapists that should supervise the CR. The demand for qualified kinesitherapies is growing, particularly as the Western population is aging. In the absence of a trained therapist, the patients loses his motivation for exercising, and they tend to become tired or bored by a monotonous training process [7]. Moreover, according to Kraal J.J. et al. the cost of rehabilitation therapies rises worldwide each year. For example, in Denmark, in 2018, motor rehabilitation therapy throughout 3 weeks following heart valve surgery exceeded 20,000 € per patient [8].

Because of these high costs, most of the patients lack the opportunity of rehabilitation therapy. While there remain a few procedures that only human therapists can undertake, many rehabilitation exercises are highly repetitive [9]. This is where robotic systems outperform the humans. They can perform the same task (s) with high precision and without exhaustion or loss of attention and concentration [10]. Nonetheless, the addressability of such robotic exoskeleton systems in delivering therapy after a cardiac open surgery or a major cardiac event is very limited [11,12,13]. The use of digital therapeutic games and virtual reality (VR) platforms as well as coupled with Artificial Intelligent agents in rehabilitation practices are increasing [14].

Despite its repeatedly proven benefits, and the clinical practice guideline recommendations, CR programs are grossly under-used. Worldwide, there is low availability of CR; only 38.8% of countries globally have CR programs [1]. We lack the strategies to increase CR availability at all levels. To surmount these difficulties systematic referral strategies, supportive public health policies and alternative models of delivery should be implemented at national and international levels.

The scope of the trial is to support motor recovery of patients following a cardiac surgery or a major cardiac event to ensure a normal active and independent life. This shall be realised by initiating CR immediately after surgery using an assistive upper-body robotic exoskeleton System enhanced with a non-immersive virtual reality exergame (CardioVR-ReTone). The aim of the clinical trial is to validate the prototype of the CardioVR-ReTone system in patients who have undergone cardiac surgery.

## 2. Materials and Methods

### 2.1. Description of the CardioVR-ReTone Exoskeleton

**Figure 1:**
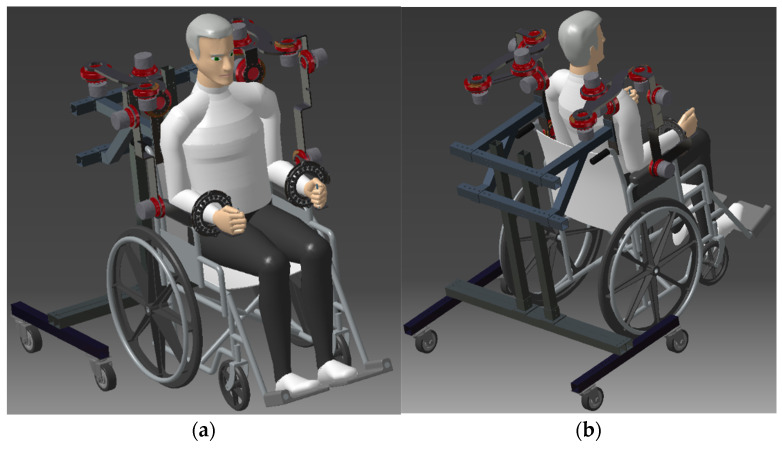
Three-dimensional model of CardioVR-ReTone robotic exoskeleton: (**a**) front view and (**b**) rear view.

The basic configuration of the CardioVR-ReTone robotic exoskeleton is highlighted in Figure 1a,b. The CardioVR-ReTone robotic exoskeleton consist of two arms, each has seven degrees of freedom (DOFs). The actuation system for each robotic exoskeleton arm consists of six DC brushed motors such as Maxon EC60fl (Maxon, Sachseln, Switzerland) that assure the necessary torque to each joint through a reducer CPU-17-M (Maxon, Sachseln, Switzerland) CPU-20-M (Maxon, Sachseln, Switzerland), CPU-25-M (Maxon, Sachseln, Switzerland)and in addition each arm contains a passive joint for facilitating the hand rotation. The CardioVR-ReTone robotic exoskeleton is attached to a floor-mounted frame, which allows you to adjust both the height and the distance between the arms as needed.

The exoskeleton has seven rotational joints making possible the following movements: flexion-extension of the shoulder, abduction-adduction of the shoulder, internal-external rotation of the shoulder, elbow flex-extension, pronation forearm-supination, flexion of the wrist extension and radial-ulnar deviation of the wrist. Redundant kinematic mechanisms facilitate flexibility in positioning and orientation due to their possession of more DOF than necessary. Thus, to increase the manoeuvrability of the CardioVR-ReTone, five DOFs are assigned to the shoulder complex, the elbow joint and the radio articular joint are each modelled by a single DOF.

**Figure 2:**
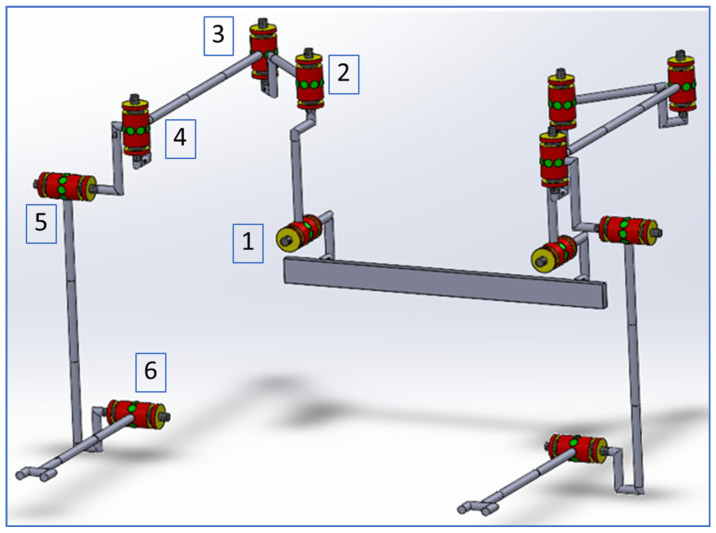
The exoskeleton kinematic scheme–motors labelled from 1 to 6.

The exoskeletal actuated joints are labelled from 1 to 6 in the order shown in Figure 2, and the passive joint that will allow the wrist radial-ulnar deviation it is not highlighted in the figure.

### 2.2. Space Requirements–Specification

The patient’s cardiac rehabilitation movements should not be restricted by the size and movement capabilities of the CardioVR-ReTone exoskeleton. The minimum space required for the safe use of the CardioVR-ReTone is 1.2 × 2 m^2^.

**Figure 3:**
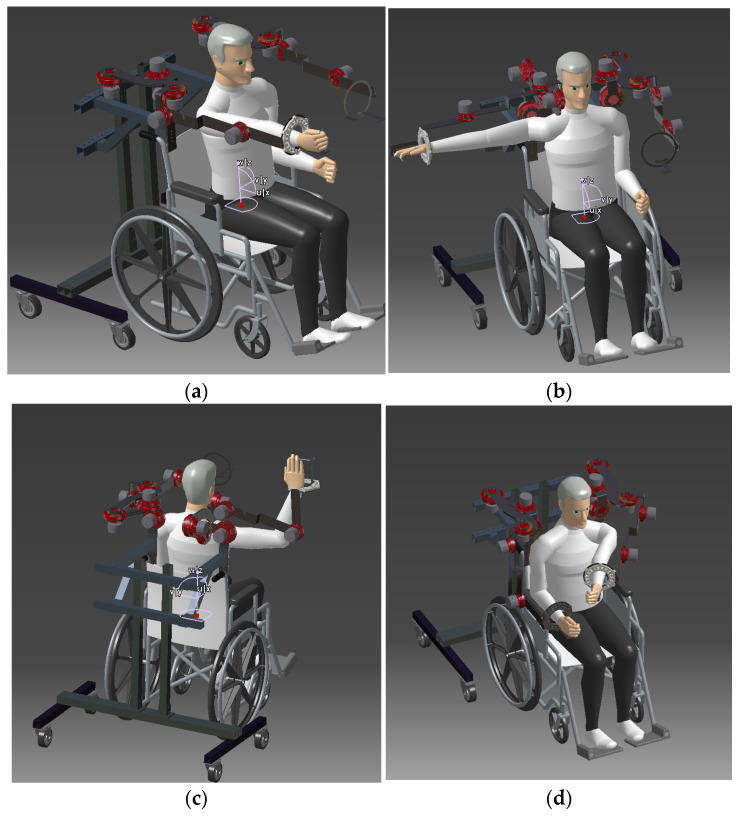
The main possible movements of the CardioVR-ReTone robotic exoskeleton. The main possible movements of the CardioVR-ReTone robotic exoskeleton: (**a**) elevation and flexion of the shoulder and arm; (**b**) elevation and extension of the shoulder and arm; (**c**) elbow flexion/extension; (**d**) adduction of the arm and forearm.

The technical characteristics (hight, width, footprint and weight) of the CardioVR-ReTone robotic exoskeleton are mentioned in Table 1. The technical characteristics are adapted to the length of the arms and to the movement capabilities. CardioVR-ReTone exoskeleton robotic exoskeleton is capable (Figure 3) to augment the necessary CR movements of the upper body.

The CardioVR-ReTone robotic exoskeleton is capable (Figure 3) to augment the necessary cardiac rehabilitation movements of the upper body.

### 2.3. Setting

#### 2.3.1. Enrolment of the Patients

The trial will be carried out at the Cardiac Surgery Department of one tertiary hospital Institute for Cardiovascular Diseases Timisoara which is a university-affiliated teaching hospital. The participants recruited will undergo cardiac surgery via a median sternotomy. Ethics approval for the study will be obtained from the Etic Committee of University of Medicine and Pharmacy (registration number 350/02.21.2019). Every volunteer will sign an informed consent. The expected number of participants is 30 (an average of five participants recruited/month, for 6 months).

#### 2.3.2. Eligibility Criteria

For the hospitalized patients are age >18 years, agreement to participate by signing informed consent, coronary artery bypass graft surgery, cardiac valve surgery, or both via median sternotomy. The exclusion criteria are insufficient Romanian-language comprehension, debilitating pathology of upper limb/shoulder, musculoskeletal system that limit exercise capacity, moderate to severe chronic obstructive pulmonary disease addiction to alcohol or drugs, symptomatic psychiatric illness (dementia, schizophrenia, acute psychosis) or severe neurological illness (stroke, Parkinson diseases).

#### 2.3.3. Recording of Demographic and Clinical Data

Demographic data: name, file number, age, sex, BMI, the length of the upper arm, blood pressure at both arms, cardiac frequency, SaO_2_, ECG, CPET for aerobic capacity recording before surgery (Table 2).

#### 2.3.4. Establishing Testing Protocol

In order to test the CardioVR-ReTone system and adjust it for post operatory rehabilitation programs a set of standardized exercises, in accordance with rehabilitations guidelines [15] is available (see the Supplementary Files).

#### 2.3.5. Testing the Exoskeleton and Recording the Results

**Figure 4:**
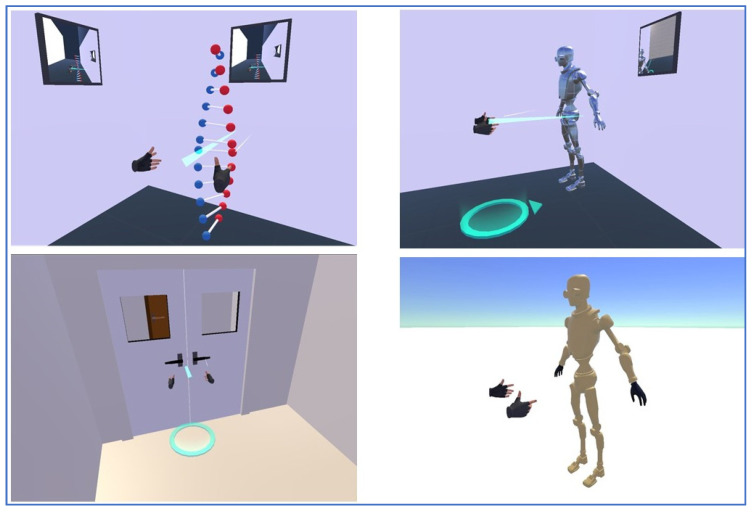
Different virtual reality (VR) games that can be used in association with the exoskeleton, such as manipulating the coloured balls, moving the robot’s arms, opening a door, controlling the android robot, in clockwise order from top-left.

All of the participants will be trained how to use the exoskeleton and the EMG electrodes will be placed following the steps provided by the producer (e.g., Delsys Trigno, Delsys Inc., Boston, MA, USA). All the patients will wear a sternal support. The test will take around 30 min. In the meantime, based on the verbal instructions of each participant, the exoskeleton will be adjusted until they feel comfortable with it and no further adjustment will be necessary. Two unblinded climatotherapists with experience in cardiovascular rehabilitation will train the patients. The movements will be recorded using Optitrack V100:R2 cameras. EMG signals will be recorded using a wireless system (e.g., Delsys Trigno, Delsys Inc., Boston, MA, USA) to quantify muscle activity. The movements will be enhanced by using a non-immersive virtual reality exergame which will be projected on a screen in front of the patient. The VR possibilities are detailed in Figure 4, and the patients can choose which game he/she prefers. The total time of the exercise and the number of repetitive cycles will be registered. Cardiac frequency and SaO_2_ will be monitored and recorded throughout the exercises. The patients’ feedback regarding pain or pressure will be constantly recorded. Pain intensity will be measured every 5 min during the test using the numerical pain assessment scale. The McGill Pain Short Form Questionnaire version 2 will be used to measure pain intensity after the test. The main reasons for terminating the physical training include: the patients’ request to stop due to severe symptoms (i.e., chest pain, shortness of breath or fatigue, palpitations), severe exercise-induced hypotension (<100/50 mmHg) or hypertension (>250/110 mmHg), signs of low cardiac output (pallor, cyanosis, cold extremities, dizziness), neurological signs (confusion, ataxia).

The sternal pain will be differentiated from angina pectoris by clinical evaluation (pain characteristics, duration, and its resolution after antalgic treatment). If the pain persisted after the physical training stopped, EKG and troponins should be measured. To prevent the angina, moderate to high-risk patients because of left ventricular dysfunction, coronary disease severity, comorbidities, ageing: will be starting at 40% of the HR. In case of asymptomatic ischemia consider 40–60% of heart rate reserve at the onset of ischemia. For the patients known with coronary artery disease, prophylactic nitroglycerine will be administered at the start of the training session.

#### 2.3.6. Appling the Clinical Protocol and Recording the Results to Validate CardioVR-ReTone System

**Figure 5:**
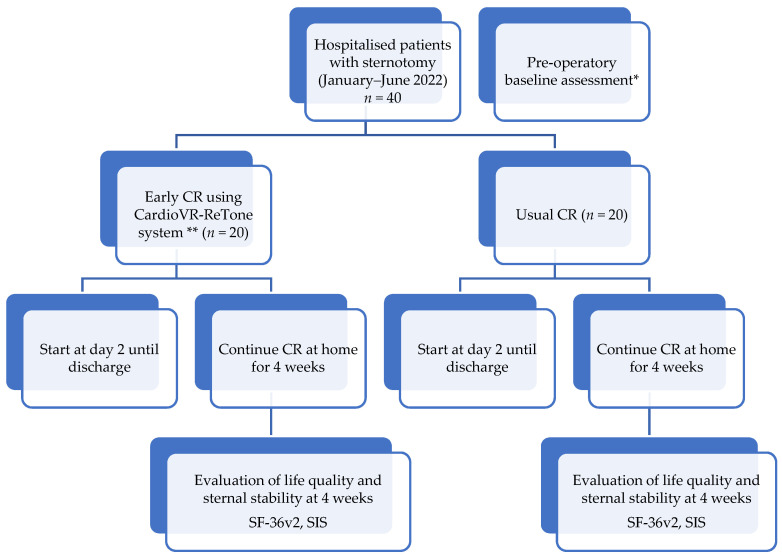
Patients’ randomisation algorithm. * Preoperative base-line evaluation consists of clinical evaluation, SaO_2_, ECG, CPET for aerobic capacity recording before surgery. ** During CR with CardioVR-ReTone, EMG signals will be registered, cardiac frequency, SaO_2_ and pain intensity will be measured with the numerical rating scale for pain at every 5 min during the test. Pain quality will be measured using the Short Form McGill Pain Questionnaire version 2.

The participants will be randomly assigned into two groups: one following CR program with CardioVR-ReTone system and another following a usual CR program (Figure 5). The patients will be allocated to early CR using the Cardio-VR-ReTone System or early CR with the usual physical training, via block randomisation on a 1:1 basis. A designated statistician will randomly allocate patients. A member of the research team will implement the randomisation sequence. The cardiac surgeons and the team members responsible for interpreting the results will be blinded to group allocation. The CR staff responsible for the physical training and the patients will not be blinded. CR will be delivered daily from day 2 after surgery until discharge. The CR program will be fully explained to both groups and will be cautiously monitored by experienced physiotherapists. Each participant will receive a printed and a standardised verbal exercise list delivered by the kinesitherapies. The patients’ feedback regarding pain or pressure will be constantly recorded. Both groups will continue exercising at home without the help of the CardioVR-ReTone system and will be clinically evaluated at 4 weeks after discharge. The Medical Outcomes Study 36-item Short Form Health Survey (SF-36v2) for evaluating the QoL and Modified Sternal Instability Scale (SIS) will be used to assess sternal instability after 4 weeks of training. Results will be recorded as described in an electronic database.

#### 2.3.7. Statistical Analysis

The primary hypothesis will be examined by analysis of variance by repeated measurements to assess postoperative changes from baseline to 4 weeks in the mobility of the arms. In all tests to be performed, a *p* value < 0.05 (with two tails) will be considered statistically significant, and confidence intervals will be reported.

## 3. Results

The results obtained will be introduced in a database, and descriptively and statistically analyzed in order to validate CardioVR-ReTone system in clinical practice. A final report will be delivered and discussed with the technical design team.

### 3.1. Outcome Measures

The primary outcome measurement is the modification of life quality at the end of the CR exercise training program. Secondary outcomes will encompass measurements of sternal stability, muscular activity (EMG signals) and pain level. Table 2 represents a framework for the schedule of outcome assessments.

### 3.2. Primary Outcome

Before the start of the CR program and after the completion of the intervention program for both groups, we will evaluate health-related quality of life by means of the Short Form Health Survey-36 Questionnaire (SF-36). The SF-36 is an assessment tool used by a multitude of research teams in the evaluation of health-related quality of life in patients with heart disease. The SF-36 questionnaire aims at two different constructs to measure health-related quality of life: the physical component and the mental component. It comprises 35 scoring items, divided into eight dimensions: physical function, physical role, emotional role, social function, mental health, general health, body pain and vitality. The outcome measurements and instruments are synthetised in Table 3.

### 3.3. Secondary Outcomes

Functional fitness will be assessed at baseline and in a continuous manner throughout the intervention program:

- skeletal muscle function assessment: the surface electromyographic activities will be recorded for upper limb muscles, using a wireless EMG system.
- cardiac frequency and oxygen saturation will be monitored and recorded for the whole duration of the CR program.

Pain intensity will be measured using SF-MPQ-2. The pain intensity scale is a validated instrument for chronic pain evaluation, including pain due to rheumatic conditions or of neuropathic origin [16]. The original version of the scale called SF-MPQ was proved to be a reliable tool to evaluate postprocedural pain in cardiac populations (α coefficients 0.75–0.83). Lovejoy et al. validated the use of the SF-MPQ-2 in older patients with chronic pain of either neuropathic and non-neuropathic origin [17]. The questionnaire comprises four subscales, and the mean of the items in each subscale is used to obtain the final score. Scores on each subscale can range from 0 to 10. The higher the score the more severe the pain [17]. Participants will choose the number that best describes their intensity of pain and related symptoms experienced during the previous week. A 0 score will be assigned if the word does not describe the participant’s pain or related symptoms. The SF-MPQ-2 is sensitive to change in chronic pain, and total and subscale scores are responsive to change. The changes are associated with patient ratings of global improvement in clinical trials [16].

## 4. Discussion

### 4.1. Types of Robotic Exoskeleton and Virtual Reality (VR) Used in Cardiac Rehabilitation (CR)

Robotic exoskeletons used in rehabilitations—so far, several robotic exoskeleton systems have been developed for the upper body extremity that have provided promising rehabilitation outcomes, but each of them has limitations. Some examples of exoskeleton structures were elegantly assessed in two recent reviews conducted by Gull et al. [18] and Qassim et al. [19]. Some of the exoskeletons useful for rehabilitation could be highlighted here: CADEN, ARMinIII, RUPERT, ReWalk, Ekso, Indego, and ALEX [19]. However, traditional exoskeletons which provide fix guidance are not adapted to the patient’s intention, thus reducing the CR efficiency. None of the models highlighted above and others from the scientific literature [20] focus on controlling the dynamics (dynamic model) of the exoskeleton. Adapting to the patient’s intention means that the exoskeletons need to be dynamically transparent and to permit the patients to take over the task whenever he wants to. The robot should have a good performance and impedance control in order to be considered dynamically transparent [21]. The ability to control force and impedance is also beneficial for implementing a novel therapeutic exoskeleton and providing a safe interaction with the patients. However, very few upper-body exoskeletons employ force and impedance-oriented actuators, such as series elastic actuators (SEAs) [20].

Virtual reality and artificial intelligence used in rehabilitation: through gaming principles and interactivity research indicated that the enjoyment of VR and AI agents in the domain of rehabilitation increased patient adherence to rehabilitation plans or willingness to stay engaged [22]. In the past decade, the number of VR technologies and VR games has increased dramatically due to available computational power and affordable digital systems [23]. In 2019, 7 million commercial head-mounted devices were sold, and the sells might reach 30 million per year by 2023 [24]. Such an explosion of environment-augmented games with emerging VR and AI is considered very useful, since they allow personalised rehabilitation plans tailored to everyone’s abilities. Moreover, the capture of natural movements enhances the motivation to perform the activities indicated by health professionals. Some of these recently developed rehabilitation games are worth mentioning: Motion Rehab AVE 3D; Gabarello for Lokomat; MobiAssist; Remission [23]. They all have in common a stimulating and interactive nature, explicit educational purpose, and offer an enriched environment of elements that support and motivate the learning of motor skills [25]. There have been many attempts during the last decade to validate new technologies in CR. A recent meta analysis conducted by Garcia-Bravo et al. showed that VR can be used as a complementary tool in different stages of CR [26]. These new technologies based on VR and video games are shown to increase adherence to CR programs. However, after analyzing 10 studies focusing on VR during CR, the authors concluded that “studies with adequate methodological quality are necessary in order to determine the technological systems, cardiac diseases, protocols and training intensity levels” [26].

### 4.2. Dissemination and Impact

The results of trials are supposed to change the way CR is conducted in sternotomy patients and also to influence future research in this domain. Research findings will published in peer-reviewed journals. As such, findings, relating to both scientific outcomes and CR service provision will be disseminated among national governing bodies and associated organizations, via newsletters and conferences in order to increase CR importance and knowledge at all levels.

The outcome of the project is split into two categories of information: (i) public information and (ii) classified information that shall be used by industrial partners only. The first category includes the results produced in the early stages of the design and manufacturing the CardioVR-ReTone system. All these results will be disseminated by following the usual rule of scientific publication. New knowledge emerging from the project will be subject to patent filing and licensing, as well as high-quality and impact paper publishing. The personal presentation of results early on the meetings of the network and the close collaboration within the network will help to disseminate results to other researchers and institutes. The research results will also be presented at seminars, workshops and international conferences for the broad scientific community and the general public. Together with the public relations departments of the partner institutions, special attention will be paid to dissemination to the general public through media, daily press, science fairs and popular science magazines in order to help to create a positive recognition of science.

To summarize the dissemination vectors for the public information produced in the project will be: (i) publications (at least three) in journals with high impact factor (about three or more)—the project’s topic and the scientific skill of the research team are solid arguments to propose this target; (ii) the use of the Open Access facility for the most important publications; (iii) publication aton-line on scientific web sites in order to make the results available to a wider scientific community; (iv) attendance at national and/or international conferences and workshops.

The second category of information includes the results obtained in the late stages of optimizing the CardioVR-ReTone system. These results will not be subject to any dissemination since they are expected to form the backbone of the new technological development.

### 4.3. Risk Prediction

The risk factors associated to the project are specific to each of the activity in terms of theirs effects and probability. The potential risks of this project are minimal, starting from the large experience of the team members in recent years. The previous collaboration on two patents proposals: “Method and laparoscopic instrument for accurate position identification of colon tumour”, OSIM, No. 2014 00821/03.11.2014 [27,28], and “Arterial puncture device for arterial blood gas sampling from radial artery and method for using the device”, OSIM, No. A00475/13.07.2017 [29] and on four research papers [5,6,27,28], prove their ability to develop and identify new research opportunities applied directly to the rehabilitation of cardiac patients. Also, the expertise of the member of the project regarding the cardiac surgery and rehabilitation of cardiac patients, a top clinical and research centre from Romania, recommended it to be a valuable partner of this project. A more realistic scenario that requires alternative planning is the lack of funds initially allocated and redistributed in the coming years. These can lead to delays in the achievement of the stages (insufficient quality) that can delay the overall and potential progress, compromising the overall quality of the result. Taking this scenario into consideration, a contingency plan is an integral part of the overall project management, fully integrated with progress monitoring, quality control and communications flow management.

Their list of risk factors associated with the project and mitigation measures are given below:

- R1: Risk (associated level of likelihood): <medium to low> project scope too narrow; changes in management/financial part. Proposed mitigation measures: (i) the project was designed broad enough to explore different potential approaches; (ii) adapt the budget/consortium structure;
- R2: Risk (associated level of likelihood): <medium> (i) poor mechanical quality of the components; (ii) insufficient details in the design; (iii) improper 3D printed components; (iv) ergonomics issues in energy transfer to human body; (v) faulty assembly the exoskeleton in test laboratory; (vi) incorrect user body size assumptions; (vii) vibrations of actuator, equipment; (viii) harmful levels of acoustic noise for the patient. Proposed mitigation measures: (i) we foresee supplementing the number of experimental investigations; (ii) re-designing the mechanical/electrical component/and/or change the manufacturing procedure; (iii) reprinting the problematic components using different extrude material; (iv) using clickable tools or cables connected for energy supply and communication; (v) reassembling the exoskeleton in the correct way; (vi) the exoskeleton will be designed to permit adjustments/connected (ISO 13482; ISO 13849); (vii) adjusting the control and redesign the linkages; (viii) using noise-absorbing materials;
- R3: Risk (associated level of likelihood): <medium>: (i) incorrect identification of requirements regarding the VR and game module; (ii) the VR game module do not meet the needs; (iii) inconsistency in VR application development. Proposed mitigation measures: (i) applying the iterative “Agile” approach in developing the VR module; (ii) redesigning the application and address the issues regarding VR module;
- R4: Risk (associated level of likelihood): <medium> relevance of data may be limited for the final goal. Proposed mitigation measures: if this is the case, new sets of investigations will be proposed based on a different medical protocol; adjustments in exoskeleton structure may be necessary;
- R5: Risk (associated level of likelihood): <medium> relevance of data may be limited for the final goal: Proposed mitigation measures: if this is the case, new sets of investigations will be proposed based on a different medical protocol; adjustments in exoskeleton structure may be necessary.

## 5. Conclusions

We have brought to attention the protocol of a randomised control trial with the aim of validating the prototype of an assistive CardioVR-ReTone system in patients who have undergone cardiac surgery. We have underlined the risk factors associated with the project and mitigation measures. Only after the feasibility of the CardioVR-ReTone system has been demonstrated using the present study protocol, do we intend to evaluate its beneficial consequences on improving cardiovascular functional capacity after cardiac surgery. We hope that implementing these novel features of the CardioVR-ReTone system, the addressability and efficacy of CR, so problematic in certain situations and especially in cardiac surgery, will be greatly facilitated, being independent of the skills and availability of the rehabilitation therapist.

## Figures and Tables

**Table 1:** Technical characteristic of CardioVR-ReTone.

CardioVR-ReTone Gouge	Dimensions
Max. exoskeleton height when in use	1610 [mm]
Max. exoskeleton height when in standby	1100 [mm]
Max. exoskeleton width when in use	1820 [mm]
Max. exoskeleton width when in standby	1000 [mm]
Max. exoskeleton footprint—length/width	1200 [mm]/800 [mm]
Maximum weight of the exoskeleton/and the support	58 kg

**Table 2:** Recordings of demographic and clinical data.

Demographic and Clinical Data	Pre-, Intra-, and Post-Surgery Details
Name, Surname, phone No. Medical file No. Birthday Sex M/F Occupation Education level Smoking history Height and weight (BMI) Date of hospitalisation and discharge BP CF SaO_2_ ECG CPET for aerobic capacity	Schedule of the surgical intervention Medical information (LVEF, NYHA classification, co-morbidities, the type of the graft) Surgical procedure (type of the procedure, elective or emergency procedure,) Intra-operatory details (method of sternal closure, cardiopulmonary bypass time, duration of the intervention, adverse events if any) Time spent in ICU (h) Pain medication (before and after surgical intervention) Other drugs (pre- and post-operative)

BMI: body-mass index, BP: blood pressure; CF: cardiac frequency; CPET: Cardiopulmonary effort test, ECG: electrocardiogram, ICU: intensive care unit, LVEF: left ventricular ejection fraction, NYHA: New York Heart Association, SaO_2_: oxygen saturation.

**Table 3:** Schedule of outcome assessments.

Measurements	Instruments
Quality of life	Short Form Health Survey-36 Questionnaire (SF-36)
Sternal stability	Modified Sternal Instability Scale
Upper arm skeletal muscle function assessment	Wireless EMG system
Pain intensity	Short Form McGill Pain Questionnaire version 2 (SF-MPQ-2)
Exercise programme	Total time of the exercise (min) and number of repetitive cycles (No.)
Cardiac response to exercise	SaO_2_, arterial pressure, cardiac frequency
Compliance/Adherence	Dropout rates (number of patients that got out of the study/total number of patients)

## Supplementary Materials

The following are available online at https://www.mdpi.com/article/10.3390/ijerph182211922/s1. Supplementary materials presenting the designed protocol for physical exercises.
