# Supplementary material for: Cardiac Rehabilitation Early after Sternotomy Using New Assistive VR-Enhanced Robotic Exoskeleton—Study Protocol for a Randomised Controlled Trial
Source: Int J Environ Res Public Health. 2021 Nov 13;18(22):11922. doi: 10.3390/ijerph182211922 (PMC8622225; doi:10.3390/ijerph182211922)
Supplement: Supplementary file 1 [file ijerph-18-11922-s001.zip › ijerph-1432829-supplementary.pdf]

Noul exoschelet robotizat cu sistem integrat de realitate virtuala pentru reabilitare cardiaca – CardioVR-ReTone -

Livrabil-R1_L1

Strategii, terapii si exerciții fizice necesare recuperării pacienților cardiaci

Contents

Scopul acestui document 3

Abrevieri și notații folosite 3

1. Procedura de realizare a sternotomiei si presupuse limitări în recuperarea poststernotomie 3

2. Strategii de recuperare cardiacă 5

3. Gimnastica respiratorie 8

4. Model program recuperator precoce 9

5. Identificarea etapelor in care este util exoscheletul 11

Referințe 11

Scopul acestui document

Scopul acestui document este de a detalia și înțelege **procedura de sternotomie în cazul intervențiilor chirurgicale cardiace** și de a evalua diverse **abordări, strategii, terapii și exerciții fizice** necesare recuperării pacienților cardiaci și formularea unor recomandări de recuperare care să se preteze a fi realizate cu ajutorul unui exoschelet robotizat.

Abrevieri și notații folosite

g.d.l./gdl	Grade de libertate
1 MET	Cheltuiala energetică a organismului in repaus, necesară susținerii funcțiilor vitale și se definește ca un consum de O2 (VO2) de 3,5 ml O2/kg/min sau aproximativ 250 ml/min pentru un organism de 70 kg.
Modelul FITT	Frecventa, Intensitate, Timp, Tip de exercițiu

1. Procedura de realizare a sternotomiei si presupuse limitări în recuperarea poststernotomie

Sternotomia mediană este principalul abord in chirurgie cardiaca, adulți si copii, fiind folosita in peste 90% din operațiile cardiace. Ea se realizează prin incizia pielii si a țesutului celular subcutanat, pentru a aborda sternul. Se prepara furculița sternala precum si xifoidul, care se secționează sau chiar se rezeca. Se identifica apoi mijlocul sternului si se marchează cu cauterul electric. Sternotomia propriu-zisa este apoi realizata cu ajutorul sternotomului electric, un instrument care este practic un fierăstrău electric pentru uz medical.

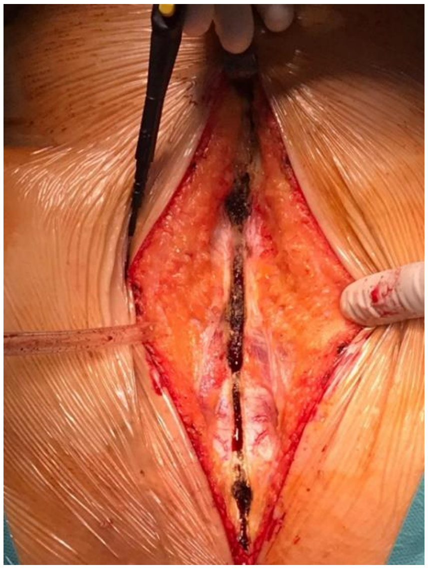

Fig. 1 Prepararea sternului

Avantajele acestei cai de abord este expunerea completa a cordului, pericardului si originii vaselor mari. La finele operației se realizează sternorafia, adică sutura marginilor sternale, cu ajutorul unor sârme, de grosimi diferite, care se trec prin stern sau prin spatiile intercostale, razant la marginea laterala a sternului. In situația in care sternul este de calitate proasta se recurge la o tehnici alternative, care stabilizează atât longitudinal cat si transversal sternul.

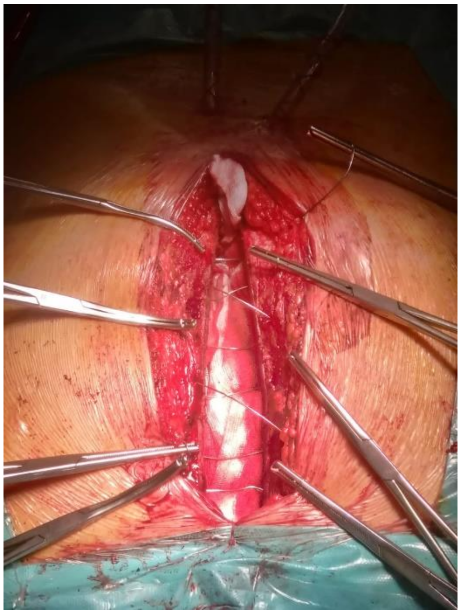

Fig. 2. Amplasarea firelor de sarma

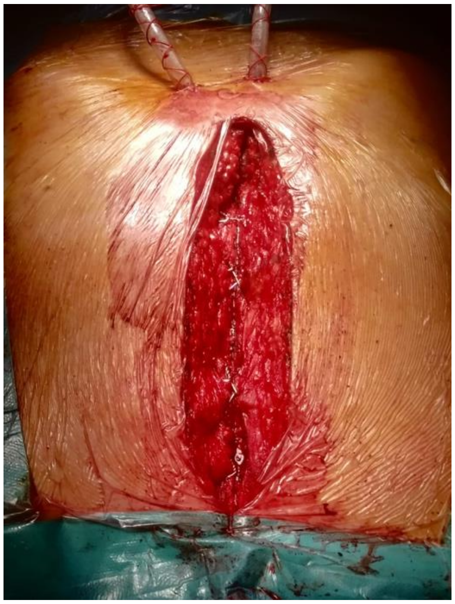

Fig. 3. Sternul fixat

Postoperator, pacienții abordați printr-o sternotomie completă vor prezenta un disconfort dureros, a cărui intensitate variază de la pacient la pacient, percepția durerii fiind extrem de individualizata. In consecința, pacienții tind sa execute mișcări respiratorii cât mai superficiale, pentru a nu induce apariția durerii prin recrutarea sternului. Pe de alta parte, pentru a favoriza vindecarea sternului, care este un os gros, pacienții sunt încurajați sa poarte un corset care fixează cele doua jumătăți sternale si împiedică deplasarea lor, de exemplu pe parcursul tusei sau a strănutului. De asemenea, sunt evitate mișcările ample, de abducție sau de ridicare deasupra nivelului capului realizate cu brațele. Acest lucru are drept urmare o recuperare deficitară a mișcărilor respiratorii precum si a jumătății superioare a corpului, cu impact asupra capacității de efort a pacientului si pierderea masei musculare.

Recent au fost puse la punct tehnici de abord miniminvaziv, care evita realizarea sternotomiei complete, tocmai pentru a evita consecințele acesteia, abordul fiind fie prin minitoracotomie laterala de 5 cm, sau prin hemisternotomie in „J".

In concluzie, consecințele sternotomiei complete sunt:

- Instabilitate sternala
- Durere la mobilizare
- Recuperare respiratorie lentă
- Pierderea masei musculare
- Ambulație dificilă

2. Strategii de recuperare cardiacă

Procesul de recuperare cardiovasculară este indicat tuturor pacienților supuși intervențiilor chirurgicale. Acesta are rol de a îmbunătății calitatea vieții și supraviețuirea pe termen lung a pacienților. Studiile recente arată că participarea la programele de recuperare cardiovasculară se asociază cu o reducere a mortalității cu 40% în cazul pacienților cu bypass aorto-coronarian, precum și refacerea funcțională și reintegrarea profesională mai rapidă ap pacienților supuși intervențiilor chirurgicale valvulare. Aceștia din urmă obțin îmbunătățiri asemănătoare pacienților cu bypass-aortocoronarian în condiții de participare la antrenament aerob, îmbunătățiri similare, indiferent de tipul și gradul de afectare valvulară.

Premisele teoretice ale instituirii precoce a programelor de recuperare pornesc de la realitatea faptului că repausul prelungit la pat poate duce la escare de decubit care se pot suprainfecta. Atrofia musculară cauzată de restricțiile postoperatorii are ca efect reducerea capacității funcționale. Pe de altă parte, exercițiu aerobic îmbunătățește funcția pulmonară, prin întărirea mușchilor respiratori, îmbunătățirea mobilității toracice și a echilibrului dintre elasticitatea pulmonară și cea toracică.

Pentru creșterea complianței la exerciții fizice se poate recomand inițierea unui program de reabilitarea cardiacă preoperator. Acest program preoperator este justificat de îmbunătățirea condiției fizice a pacientului și de familiarizarea acestuia cu tipurile de exerciții specifice recuperării ți cu noile tehnologii utilizate în reabilitare (tehnici de virtual-reality și de mișcări pasive și active coordonate de exoskeleton robotizat) care vor fi detaliate în secțiune de mai jos.

Procesul de recuperare cardiovasculara a pacienților supuși intervențiilor chirurgicale cardiace cuprinde 3 faze. Faza I intraspitalicească, faza II de recuperare la domiciliu si faza III de consolidare a rezultatelor obținute in fazele anterioare. In acest raport ne vom concentra asupra fazei I de recuperare intraspitalicească. Însușirea cât mai rapid de către pacient a unei suite de comportamente și exerciții fizice pe care să le poată aplica și automonitoriza la domiciliu reprezintă cheia în recuperarea postoperatorie.

Faza I de reabilitare după intervențiile chirurgicale cardiace se inițiază în primele zile postoperator pe secția de terapie intensivă și se continuă post-terapie intensivă în măsura în care condiția clinică a pacientului o permite. Această fază este importantă in prevenția complicațiilor, permite o recuperare mai rapidă și este recomandată celor care nu prezintă contraindicații pentru exerciții fizicele (insuficiență cardiacă severă NYHA IV).

Obiectivele fazei I:

- evitarea decondiționării fizice cauzată de repausul prelungit,
- instruirea pacientului in ceea ce privește tehnicile de efectuare a exercițiilor și
- educarea cu privire la măsurile necesare modificării stilului de viață.

Faza I se desfășoară pe parcursul perioadei de internare și presupune mai multe componente de bază, precum:

- exercițiile respiratorii menite să asigure o ventilație corectă și să mobilizeze musculatura respiratorie – ***gimnastică respiratorie***
- ambulația progresivă și exercițiile fizice zilnice menite să prevină atrofia musculară și complicațiile decubitului dorsal prelungit – ***mers și kinetoterapie pasivă și activă*** și

- reluarea capacității de autoîngrijire deplasare independentă.

Strategia recuperatorie din faza I reprezintă subiectul principal al raportului de față. Ea cuprinde 3 părți:

- ziua 1 postoperator – ***mobilizarea și gimnastică respiratorie***
    - mobilizarea pasivă și activă a membrelor
    - ridicarea la marginea patului în poziție sezândă
- zilele 2-6 postoperator – ***mobilizarea, gimnastica respiratorie, deplasări progresive și exerciții fizice zilnice cu dificultate progresivă***
    - ambulația inițială se efectuează asistat, pe distante de 150-200 m în ziua 2
    - ambulația independentă se poate efectua din ziua 3
- zilele 5 - 8 (sau până la externarea pacientului) – ***consolidarea activităților din zilele 2-5 și*** planificarea programului de exerciții fizice pentru faza II (postspitalizare)
    - pacientului i se oferă un program de exerciții respiratorii și fizice, pe care să le poată aplica după externare și informații despre boala cardiacă proprie și despre modificarea factorilor de risc.

Metodologia fazei I poate urma modelul FITT:

- Frecvența exercițiilor – zilnic din prima zi postoperator
- Intensitatea – monitorizarea intensității exercițiilor se face prin 3 parametri de bază:
    - Starea clinică
    - Frecvența cardiacă (FC) de antrenament care nu trebuie să depășească și nici să nu fie inferioară frecvenței prestabilite (65-75% din FC maximală calculată FCmax= 220-vârsta)
    - Scala de evaluare a efortului (Scala Borg) împărțită în 20 de grade (Tabelul 1)
    - **La pacienții postoperator se recomandă programe de intensitate mică cu creștere progresivă până la un maxim de 2-4 METs atins în ultima zi de internare, cu o FC de 65-75% din FC maximală calculată și un nivel al exercițiului care este egal cu o evaluare a eforului perceput de 7-8 (foarte ușor) pe scara Borg până la 10-12 (destul de ușor) la externare.**
    - Înainte de externare se recomandă test de efort submaximal, care va permite evaluarea capacității fizice a pacientului și prescrierea indicațiilor de antrenament fizic în faza II.
- **Timpul – fiecare ședință să fie alcătuită din 3 etape (durata totala 30-40 minute)**
    - **etapă de încălzire cu durata de 5-10 minute,**
    - **etapă de antrenament propriu-zis de cel puțin 20 minute și**
    - **etapă de recuperare de 5-10 minute.**
- **Tipul exercițiului – ca principiu antrenamentul trebuie să utilizeze grupe musculare mari și să includă exerciții aerobe**

Faza II Spital de Recuperare

Faza II presupune recuperarea cardiovasculară propriu-zisă începută la aproximativ 4-6 săptămâni post-operator, după vindecarea plăgii sternale.

Obiectivele fazei II:

- Îmbunătățirea performanței cardiace
- Recuperarea capacității de efort

Tabelul 1. Scara Borg de măsurare a efortului maxim perceput (RPE)

Scara	Măsurarea
6-8	Foarte, foarte ușor
9-10	Foarte ușor
11-12	Destul de ușor
13-14	Cumva greu
15-16	Greu
17-18	Foarte greu
19-20	Foarte, foarte greu

Se inițiază în centrele de recuperare cardiovasculară și sunt continuate la domiciliu, având o durată de aproximativ 8-12 săptămâni. Programele includ în general ședințe de exerciții fizice efectuate sub supraveghere medicală/monitorizare EKG și ședințe de consiliere medicală cu privire al aderența/complianța la tratament și controlul factorilor de risc.

Ședințele de antrenament fizic din faza II sunt obligatoriu precedate de test de efort submaximal, care permite evaluarea capacității fizice inițiale a pacientului. Metodologia fazei II urmează, de asemenea, modelul FITT.

- Frecvența exercițiilor – exerciții fizice de 3-5 ori/săptămână
- Intensitatea – monitorizarea intensității exercițiilor se face prin 2 parametri de bază:
  - La internare se recomandă test de efort submaximal, care va permite evaluarea capacității fizice și prescrierea indicațiilor de antrenament fizic în faza II.
  - **Frecvența cardiacă (FC) de antrenament** care nu trebuie să depășească și nici să nu fie inferioară frecvenței prestabilite (70-80% din FC maximală calculată FCmax= 220-vârsta)
  - **Scala de evaluare a efortului (Scala Borg)** împărțită în 20 de grade (Tabelul 1)
  - Se recomandă programe de intensitate medie cu creștere progresivă până la un maxim de 3-6,5 METs, cu o FC de 70-80% din FC maximală calculată și un nivel al exercițiului care este egal cu o evaluare a eforului perceput pe scara Borg de până la 12-13, deoarece peste aceasta limita efortul se desfășoară în anaerobioză, devenind dăunător pt bolnav.
- Timpul – fiecare ședință să fie alcătuită din 3 etape (durata totala 30-40 minute)
  - etapă de încălzire cu durata de 5-10 minute,
  - etapă de antrenament propriu-zis de cel puțin 20 minute și
  - etapă de recuperare de 5-10 minute.
- Tipul exercițiului – ca principiu antrenamentul trebuie să utilizeze grupe musculare mari și să includă exerciții aerobe

In timp, majoritatea pacienților pot crește treptat intensitatea și frecvența exercițiilor fizice, pe măsura adaptării la efort. Exercițiile benefice pot fi încorporate în rutina zilnică, alături de celelalte elemente ale unui stil de viață sănătos

3. Gimnastica respiratorie

Gimnastica respiratorie cuprinde mai multe exerciții de respirație ușor de învățat și folosit. Mai jos am expus instrucțiunile unui exercițiu de respirație și am cuprins 3 seturi de astfel de exerciții, care sunt folosite în mod curent la ICVB Timișoara pentru pacienții postintervenție chirurgicală cardiacă.

Exercițiu de respirație (instrucțiuni)

- Așezați-vă în poziție verticală
- Puneți o mână sus pe abdomen, la capătul inferior al toracelui, respirați normal și simțiți-vă mâna mișcându-se spre exterior, apoi expirați normal și simțiți-vă mâna mișcându-se spre interior
- Respirați relaxat timp de aproximativ un minut.
- Inspirați profund, păstrându-vă umerii relaxat
- Țineți respirația timp de 3 secunde apoi expirați
- Repetați acest lucru de 4 ori și apoi reveniți la respirația relaxată
- Completați 3 seturi de 4 respirații profunde la fiecare 30 de minute
- Tușiți și curățați-vă căile respiratorii la nevoie

EXERCIȚIUL 1: - efectuat în ***POZIȚIE ȘEZÂND***

1) Plasați o mână pe abdomen deasupra buricului. Cealaltă mână puneți-o pe piept în dreptul inimii. Închideți ochii și trageți aer adânc. Care mână se mișcă?
2) Lăsați abdomenul să se umfle mai mult în timpul inspirului.
3) În timp ce inspirați repetați în gând: SIMT CUM AERUL INTRĂ ÎNĂUNTRU. În timp ce expirați repetați in gând: SIMT CUM AERUL IESE AFARĂ.
4) Încercați să faceți expirul egal cu dublul inspirului.

Exemplificare:

*- Trageți aer până numărați la 3, dați-l afară până numărați la 6 – **repetați de 7 ori**. Apoi: Trageți aer până numărați la 4, dați-l afară până numărați la 8 – **repetați de 7 ori**.*

! Încercați sa creșteți numărul în funcție de capacitatea plămânilor.

! Exersați dimineața și seara câte 10 minute cu geamul deschis, daca nu este prea frig. Dacă amețiți, OPRIȚI-VĂ!

EXERCIȚIUL 2: - efectuat în ***POZIȚIE ȘEZÂND***

1) La mâna dreaptă, puneți degetul arătător și pe cel mijlociu în palmă, apoi plasați degetul mare pe nara dreaptă, iar inelarul și degetul mic pe nara stângă.
2) Lăsați închisă nara dreaptă și trageți aer pe nara stângă până numărați la 8.
3) Închideți nara stângă cu inelarul, dați drumul la degetul mare și scoateți aerul pe nara dreaptă până numărați la 8.

4) După ce ați scos tot aerul, trageți aer tot pe nara dreaptă până număraţi la 8. Apoi închideţi nara dreaptă cu degetul mare, daţi drumul la inelar şi scoateţi aerul pe nara stângă până numaraţi la 8 din nou.
5) **Continuaţi 10 minute**.
6) **Repetaţi de 2 ori pe zi, dimineaţa şi seara**.
7) După ce vă descurcaţi bine cu tehnica aceasta, puteţi trece la exerciţiul 3.

EXERCIŢIUL 3: - efectuat în ***POZIŢIE ŞEZÂND***

1) La mâna dreaptă puneţi degetul arătător şi pe cel mijlociu în palmă, apoi plasaţi degetul mare pe nara dreaptă, iar inelarul şi degetul mic pe nara stangă.
2) Lăsaţi închisă nara dreaptă şi TRAGEŢI AER PE NARA STÂNGĂ până număraţi la 2.
3) Închideţi ambele nări şi ţineţi respiraţia până număraţi la 8.
4) Daţi drumul la degetul mare şi DAŢI AFARĂ PUTERNIC AERUL PE NARA DREAPTĂ până număraţi la 4.
5) **Repetaţi exerciţiul începând cu trasul aerului pe nara dreaptă, tot in ritm de 2:8:4.**
6) **Nu vă forţaţi! Învăţaţi exerciţiul cu răbdare, gradat.**
Acest exerciţiu calmează mintea, înlătura oboseala şi creşte energia vitală a organismului.

4. Model program recuperator precoce

Ziua 1

Gimnastica respiratorie
Tapotaj
Poziționarea pacientului in șezând la marginea patului.
Mișcări activo-pasive(active ajutate) sau active ale articulațiilor :

- Mișcări de flexie- extensie, pronatie, supinatie din articulatia pumnului (Fig.4). Mișcări de flexie-extensie adductie, abductie si opozitie din articulațiile mâinii (Fig.4)

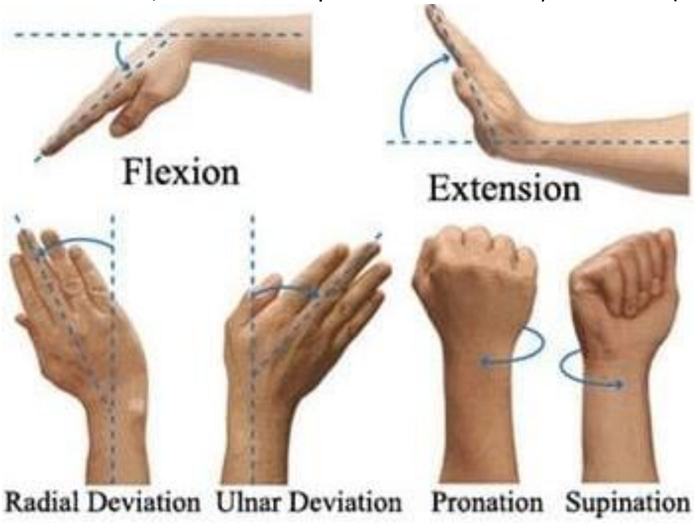

Fig. 4. Exerciții de flexie- extensie, pronatie, supinatie din articulatia pumnului

! Fiecare exercitiu in parte se executa intr-un numar de 5 repetari, o singura data pe zi.

- Flexie, abductie, aductie si rotatie interna si externa din articulatia soldului
- Flexie si extensie din articulația genunchiului
- Mișcări din articulația gleznei in toate axele si planurile
- Miscari de flexie -extensie din articulatia metatarso-falangiana

Ziua 2-3

Ridicarea pacientului in ortostatism
Miscari de schimbare a greutății de pe un picior pe celălalt
Mers in jurul patului, mers in salon, mers la toaleta
Exercitiile din ziua 1 la care se adaugă mișcări de flexie si extensie cot cu flexie cu adductie< 90 grade (Fig.5). **Mișcarea alternativă si nu simultana a membrelor superioare!**

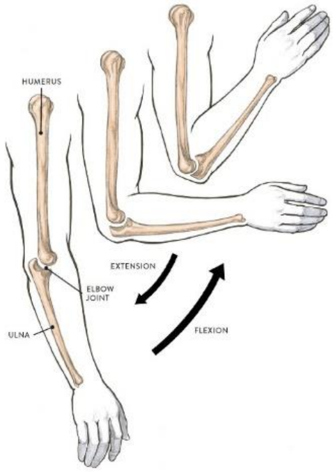

Fig. 5. Exerciții de flexie si extensie cot cu flexie cu adductie< 90 grade

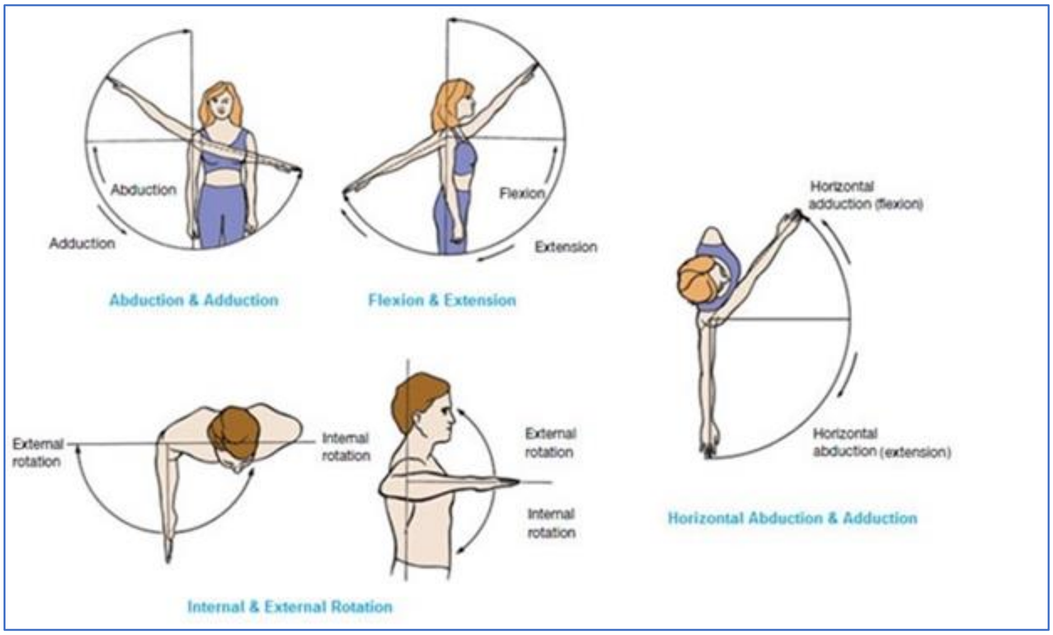

Figura 6. Exerciții de flexie umar 0-90 grade si abductie umar< 90 grade

Ziua 4-6

Mers pe coridor 20-30 m.
Urcat coborât 3-5 trepte
Exercițiile fazelor anterioare la care se adaugă flexie umar 0-90 grade si abductie umăr< 90 grade (Figura 6). **Mișcarea alternativă si nu simultana a membrelor superioare!**

5. Identificarea etapelor in care este util exoscheletul

Recuperarea post-intervențională în **faza 1 si faza 2**.
Mișcări de flexie și extensie cot cu flexie cu aducție < 90 grade (Fig.5).
Exerciții de flexie umăr 0÷90 grade si abducție umăr < 90 grade (Fig. 6).

Referințe

1. Dumitru Zdrenghea, Dana Pop, Florin Mitu Cardiologie preventiva si recuperare cardiovasculara - Editura Clusium 2020

2. Patel DK, Duncan MS, Shah AS et al. Association of cardiac rehabilitation with decreased hospitalization and mortality risk after valve heart surgery. JAMA Cardiol 2019; 4(12):1250-1259

3. Piepoli MF, Abreu A, Albus C, Ambrosetti M et al. Update on cardiovascular prevention in clinical practice: A position paper of the European Association of Preventive Cardiology of the European Society of Cardiology. Eur J Prev Cardiol. 2020;27(2):181-205. doi: 10.1177/2047487319893035

4. Ambrosetti M, Abreu A, Corrà U et al. Secondary prevention through comprehensive cardiovascular rehabilitation: From knowledge to implementation. 2020 update. A position paper from the Secondary Prevention and Rehabilitation Section of the European Association of Preventive Cardiology. Eur J Prev Cardiol. 2020 30:2047487320913379. doi: 10.1177/2047487320913379
